# Mapping ^18^F-FDG Kinetics Together with Patient-Specific Bootstrap Assessment of Uncertainties: An Illustration with Data from a PET/CT Scanner with a Long Axial Field of View

**DOI:** 10.2967/jnumed.123.266686

**Published:** 2024-06

**Authors:** Qi Wu, Fengyun Gu, Liam D. O’Suilleabhain, Hasan Sari, Song Xue, Kuangyu Shi, Axel Rominger, Finbarr O’Sullivan

**Affiliations:** 1 Department of Statistics, School of Mathematical Sciences, University College Cork, Cork, Ireland;; 2 Advanced Clinical Imaging Technology, Siemens Healthcare AG, Lausanne, Switzerland; and; 3 Department of Nuclear Medicine, Bern University Hospital, University of Bern, Bern, Switzerland

**Keywords:** dynamic PET studies, kinetic mapping, image-domain bootstrap, uncertainty assessment, compartment model

## Abstract

The purpose of this study was to examine a nonparametric approach to mapping kinetic parameters and their uncertainties with data from the emerging generation of dynamic whole-body PET/CT scanners. **Methods:** Dynamic PET ^18^F-FDG data from a set of 24 cancer patients studied on a long-axial-field-of-view PET/CT scanner were considered. Kinetics were mapped using a nonparametric residue mapping (NPRM) technique. Uncertainties were evaluated using an image-based bootstrapping methodology. Kinetics and bootstrap-derived uncertainties are reported for voxels, maximum-intensity projections, and volumes of interest (VOIs) corresponding to several key organs and lesions. Comparisons between NPRM and standard 2-compartment (2C) modeling of VOI kinetics are carefully examined. **Results:** NPRM-generated kinetic maps were of good quality and well aligned with vascular and metabolic ^18^F-FDG patterns, reasonable for the range of VOIs considered. On a single 3.2-GHz processor, the specification of the bootstrapping model took 140 min; individual bootstrap replicates required 80 min each. VOI time-course data were much more accurately represented, particularly in the early time course, by NPRM than by 2C modeling constructs, and improvements in fit were statistically highly significant. Although ^18^F-FDG flux values evaluated by NPRM and 2C modeling were generally similar, significant deviations between vascular blood and distribution volume estimates were found. The bootstrap enables the assessment of quite complex summaries of mapped kinetics. This is illustrated with maximum-intensity maps of kinetics and their uncertainties. **Conclusion:** NPRM kinetics combined with image-domain bootstrapping is practical with large whole-body dynamic ^18^F-FDG datasets. The information provided by bootstrapping could support more sophisticated uses of PET biomarkers used in clinical decision-making for the individual patient.

## Visual Abstract

**Figure:**
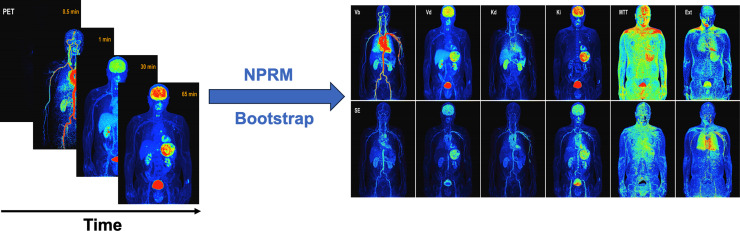

High-resolution dynamic whole-body PET scanning enhances the ability to map metabolic characteristics of tissue, particularly in the context of cancer. The current focus has been on dynamic PET studies with ^18^F-FDG using the well-established Huang–Sokoloff 2-compartment (2C) modeling framework (1–3). Although 2C modeling has had widespread application in PET imaging, far beyond the brain setting in which it was developed, the biochemical understanding of the transporters involved in the metabolism of ^18^F-FDG and their distribution across normal and cancerous tissues has evolved in the years since the Huang–Sokoloff construct was proposed (4–7). The temporal and spatial resolutions of emerging scanners have transformed the ability to objectively assess the accuracy of the 2C framework to represent ^18^F-FDG time-course data across the diverse tissues encountered in the human body. In this context, the assessment of ^18^F-FDG kinetics based on more flexible nonparametric analysis approaches (8,9) may be necessary. The most recent implementation of the nonparametric voxel-level analysis scheme (9) is particularly efficient, largely because of an extensive reliance on quadratic programming techniques, and its nonparametric aspect provides an ability to apply an image-domain bootstrapping process for evaluation of statistical uncertainties in derived kinetic maps and associated biomarkers (10,11). Uncertainties in diagnostic information recovered from PET scans could augment decision-making for individual patients that is based on complex nonlinear radiomic metrics derived from a kinetic map.

The volume of data produced by a dynamic ^18^F-FDG PET study on a state-of-the-art scanner with a long axial field of view (FOV) is a practical computational challenge for voxel-level analysis of kinetics. The bootstrap uncertainty assessment requires that comprehensive voxel-level analyses be applied to multiple simulated datasets, each created to match the full character and extent of the original data. This significantly adds to the computational challenge involved.

The work here uses a series of dynamic ^18^F-FDG data acquired on a long-axial-FOV scanner (2) to investigate the approach. Apart from the demonstration of the practical feasibility of kinetic mapping with uncertainty evaluation, the analysis allows regional comparisons between nonparametric and 2C modeling results in terms of both derived kinetics and accuracy of data representation.

## MATERIALS AND METHODS

An extended materials and methods description is provided in the supplemental materials (supplemental materials are available at http://jnm.snmjournals.org) (12).

### Patient Scans and Volumes of Interest (VOIs)

The data considered arise from a set of 24 patients with different types of cancer who participated in an institutionally approved ^18^F-FDG PET/CT study at Bern University Hospital (KEK 2019-02,193). Details of the study were reported previously (2). In summary, PET scanning was conducted on a Biograph Vision Quadra scanner (Siemens) with a 106-cm axial FOV and a nominal in-plane resolution of 3.3 mm in full width at half maximum (13). Data were acquired in list mode starting 15 s before an intravenous bolus injection of ^18^F-FDG (with activity of ∼3 MBq/kg of patient weight) to the left or right arm, followed by flushing with 50 mL of saline solution. The plasma glucose level was measured for each patient. Emission data were acquired for 65 min and binned into 62 contiguous time frames with durations of 2 × 10 s, 30 × 2 s, 4 × 10 s, 8 × 30 s, 4 × 60 s, 5 × 120 s, and 9 × 300 s. Images were reconstructed with a voxel size of 1.65 × 1.65 × 1.65 mm^3^. Low-dose CT scans (voltage, 120 kV; tube current, 25 mA; CARE Dose4D and CARE kV [Siemens]) were acquired as part of the examinations. The CT images were reconstructed with a voxel size of 1.52 × 1.52 × 1.65 mm^3^.

Automated segmentation algorithms based on CT and PET were used to define VOIs corresponding to several tissue structures, including gray and white matter in the brain, liver, lungs, kidneys, spleen, and bones (2). A further set of 49 VOIs corresponding to tumor tissue was identified by an experienced nuclear medicine physician. Finally, a VOI placed in the descending aorta was used to define the whole-blood arterial input function (AIF) used for kinetic analyses (2). Further scanning and study protocol details are available in the supplemental materials.

### Parametric Imaging Techniques

#### Tissue Residue and Kinetic Parameters

**FIGURE 1.**
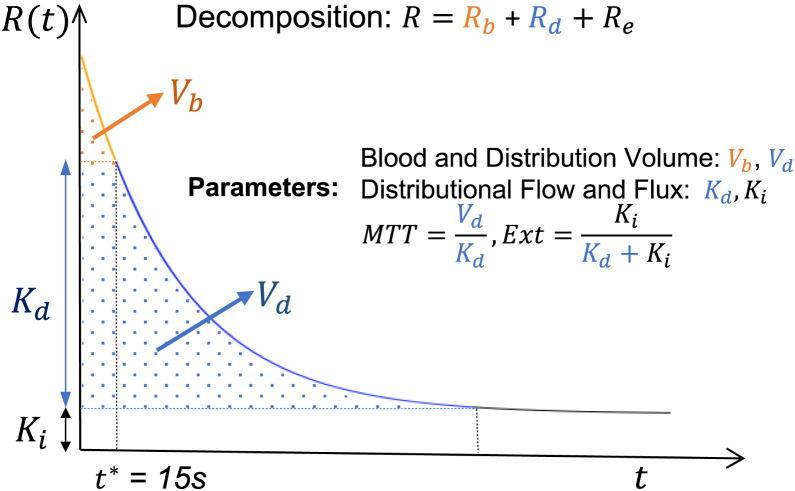
Meier–Zierler tissue residue (*R*) with decomposition into vascular (*R*_b_), in-distribution (*R*_d_), and extracted (*R*_e_) components. Decomposition was used to define indicated metabolic parameters. MTT = mean transit time; Ext = extraction fraction.

When the Meier–Zierler (14) formalism is followed, the analysis assumes the PET-measured time course for a tissue region is represented as a convolution between the local AIF, *C*_p_, and the regional tissue residue function. Kinetic parameters are defined in terms of this residue (Fig. 1). Large-vessel vascular blood and distribution volumes (*V*_b_ and *V*_d_, respectively) are evaluated as areas under the tissue residue. The apparent rate of retention or flux (*K_i_*) of the tracer, measurable by PET over the scan duration, is the height of the residue at the end of the acquisition period. Also, the mean transit time of the tracer in the tissue and extraction fraction are defined as ratios of amplitude and integral measurements. A variety of approaches might be used to approximate the residue: a nonparametric method is used here. Patlak analysis uses a constant residue (15). Compartmental model forms, for example, the 1-compartment Kety–Schmidt (16) model for water and the 2C Huang–Sokoloff (17) model for ^18^F-FDG in the brain, represent residues by positive linear combination of exponentials. In the 6-parameter 2C model, there is additive adjustment for an arterial signal. By adding a sharp residue element to the 2-exponential form, a Meier–Zierler residue is also available for this model. This allows residue-defined metabolic parameters for the extended compartmental model to be evaluated via the decomposition shown in Figure 1 (18). Supplemental materials provide a review of how Meier–Zierler residue parameters link with rate constants in the 2C model.

#### Nonparametric Residue Mapping (NPRM) of Kinetics

NPRM approximates the voxel-level residue by the positive linear sum-of-basis elements that have been selected by a cross-validation–guided analysis of a comprehensive collection of time courses produced by segmentation of all the available data in the study (10,18). Individual basis elements are of the form ${\text{μ}}_{k}\left(t\right)=\int_{0}^{t}R_{k}\left(s\right)C_{p}\left(s-\Delta_{k}\right)\text{d}s$ for *k* of 1, 2,…, *K.* Here, *R_k_* is the basis element residue and Δ*_k_* is its associated delay factor. Note that cross validation is used to select the number of basis elements (*K*). Given the basis set, PET-measured voxel-level time-course data over the available set of *J* time frames, $\{z\left(t_{j}\right),j=1,2,\ldots ,J\}$, is expressed as

$$z\left(t_{j}\right)={\text{α}}_{1}{\text{μ}}_{1}\left(t_{j}-\text{δ}\right)+\ldots +{\text{α}}_{K}{\text{μ}}_{K}\left(t_{j}-\text{δ}\right)+\text{є}(t_{j}).$$ Eq. 1

Here, δ and (α_1_, α_2_,…, α*_K_*) are the unknown voxel-level delay and basis-amplitude parameters, respectively, and є(*t*) represents (random) model error. A weighted least-squares criterion, with weights proportional to the product of the frame duration and the decay correction factor used to convert raw counts to decay-corrected tracer activity, is used for optimization of the unknown parameters. For any delay, the optimal set of α coefficients is found by quadratic programming. A crude grid search is used to optimize delay (10).

#### Bootstrap Assessment of Uncertainty

**FIGURE 2.**
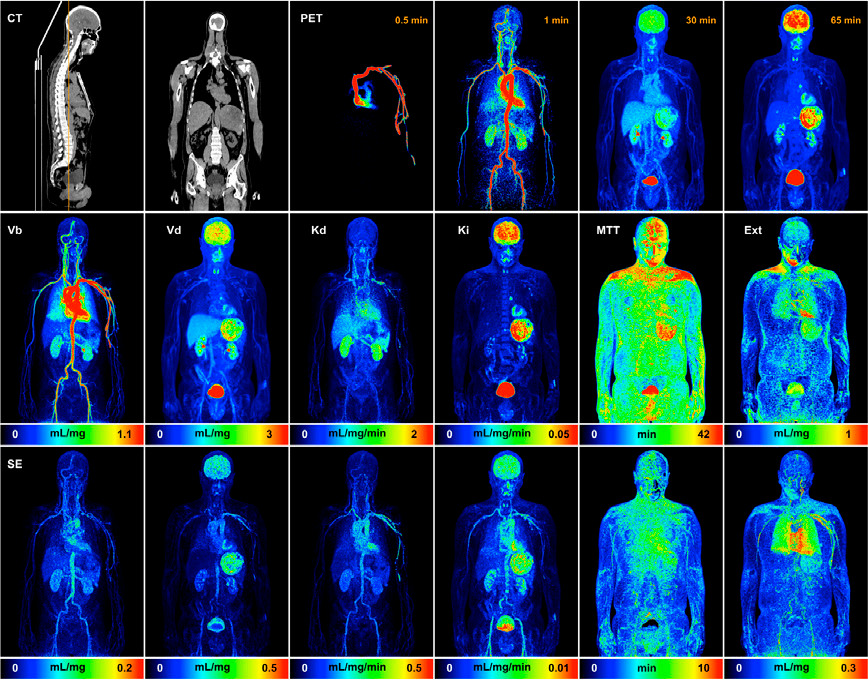
MIP maps of NPRM kinetic parameters and associated SE. SEs are based on SD of MIP results for each of 25 bootstrap replications. Top row shows CT images for selected cross sections through volume and PET MIP maps at indicated times. *K* = 9 basis elements were determined for data by NPRM methodology. MTT = mean transit time; Ext = extraction fraction.

Model residuals across *N* voxels and *J* time frames, $\{z_{i}\left(t_{j}\right)-{\hat{z}}_{i}\left(t_{j}\right),i=1,\ldots ,N;j=1,2,\ldots ,J\}$, are used to construct an image-domain data generation process (DGP) for bootstrapping. The DGP generates data according to

$$z_{i}^{*}\left(t_{j}\right)={\hat{z}}_{i}\left(t_{j}\right)+{\text{є}}_{i}^{*}(t_{j}),$$ Eq. 2

where $\hat{z}\left(t_{j}\right)={\hat{\alpha }}_{1}{\text{μ}}_{1}\left(t_{j}-\hat{\delta }\right)+\ldots +{\hat{\alpha }}_{K}{\text{μ}}_{K}\left(t_{j}-\hat{\delta }\right)$ and the simulated error process, ϵ*, mimic the stochastic character of analysis residuals. Analysis of bootstrapped datasets arising from the DGP leads to a set of bootstrapped kinetic parameter values at each voxel. The SD of these values estimates the voxel-level SE of the kinetic parameter. Similarly, the SEs for more complex quantities, such as the maximum-intensity projection (MIP) for a kinetic map, are created as the SD of the bootstrapped MIPs of the kinetic parameter (Fig. 2). Numeric studies (10,11) have shown that image-domain DGP bootstrapping matches the accuracy of the much more computationally intensive list-mode bootstrapping approach of Haynor and Woods (19).

The number of bootstrap simulations impacts the accuracy of the SEs it produces (20); this is discussed in the supplemental materials.

### Statistical Analysis

NPRM kinetic analysis with 25 bootstrapped simulations is evaluated for each of the studies in the series. Results are examined in 4 separate ways. Technical details with formulas are in the supplemental materials.

#### Representation of VOI Time-Course Data

Mean VOI time-course data are compared with the corresponding mean VOIs of the fitted voxel-level time courses, $\hat{z}\left(t_{j}\right)$, in Equation 2. Mean VOI time-course data are also analyzed using the nonparametric model and the Huang–Sokoloff 2C model including a fractional *V*_b_ and delay of the AIF. To facilitate fitting, a wide range of delays of ±5 min is allowed in the NPRM and 2C analysis of the VOI time-course data. The Broyden–Fletcher–Goldfarb–Shanno algorithm as implemented in the optim function in R (R Foundation for Statistical Computing) is used for optimization of the 2C model; more details are shown in the supplemental materials and Supplemental Figure 1.

Results of alternative analyses for a sample case are presented graphically. Formal comparisons are focused on the weighted-residual-sums-of-squares misfit achieved with alternative analyses. The mean relative difference between alternative representations of VOI time-course data and the associated SD is evaluated for each VOI type considered.

#### VOI Kinetics

Means and SDs of VOI-averaged NPRM kinetics are reported for each VOI type. Kinetics based on nonparametric and 2C analyses of VOI mean time-course data are similarly summarized. Deviations between alternative VOI kinetic values are summarized, and their statistical significance is evaluated using the paired Wilcoxon test.

#### DGP Model

The bootstrap DGP is expressed in more detail as

$$z_{i}^{*}\left(t_{j}\right)={\hat{z}}_{i}\left(t_{j}\right)+{\hat{\text{σ}}}_{e}{\hat{\text{ψ}}}_{i}{\hat{\text{φ}}}_{j}{\text{є}}_{i}^{*}(t_{j}),$$ Eq. 3

where the random errors, ${\text{є}}_{i}^{*}(t)$, are in units of SD and ${\hat{\sigma }}_{e}$ is an overall scale of the model error. In Equation 3, the factors ${\hat{\psi }}_{i}$ and ${\hat{\varphi }}_{j}$ are scale-free quantities representing the relative uncertainty across voxels (*i*) and time frames (*j*). As the PET-measured activity scales with dose, the DGP error scale (${\hat{\sigma }}_{e}$) should also scale with dose; this is examined graphically. The overall axial pattern variation is described by the scale factor ${\hat{\psi }}_{i}$. In a uniform cylindric phantom, this has a familiar U-shaped pattern related to scanner sensitivity (10). With a patient in the scanner, the distribution of activity and attenuation is far from uniform. Physiologic patient motions, such as breathing, may also impact axial variation. Skewness is a key feature of iteratively reconstructed PET data. A histogram of scaled residuals shows how the DGP captures this aspect. After adjustment for spatial scale factors, the 3-dimensional power spectrum of the normalized residual process provides insight into the effective resolution of the scanning. Coordinatewise autocorrelation functions associated with the spectrum give insight into the actual resolution of the scanner. Again, physiologic movements may well lead to the actual resolution’s deviating from what might be predicted on the basis of static phantom measurements.

#### SEs of VOI Kinetics

In theory, uncertainty in parameters recovered by kinetic model fitting should be proportional to the scale of the residual model error, but it may also be a function of the relevant sensitivity matrix for the model. We examine the relation between the bootstrap assessment of mean VOI kinetic SEs and suitable explanatory factors including the weighted-residual-sums-of-squares fit of the VOI and the mean VOI kinetic values. For each kinetic parameter, linear regression analysis on a logarithmic SE scale is applied. Adjustment of this regression analysis based on the VOI type and the kinetics are explored. Regression predictions of SEs are graphically compared with the true. Correlation values are also summarized.

## RESULTS

### Illustration

Sample kinetic MIP maps with associated SEs obtained using the NPRM technique and bootstrapping are shown in Figure 2. A video of all coronal MIP maps is provided as Supplemental Video 1. Note that the dataset is the same as that used in a previous report (2). The results are of high quality and are well aligned with the vascular and metabolic ^18^F-FDG patterns expected for key organ structures such as the brain, liver, kidneys, spleen, etc. (2). The uncertainties of *V*_b_, *V*_d_, distribution flow (*K*_d_), and *K_i_* are generally higher for regions with larger magnitudes for the kinetic variable. This is perhaps related to the fact that these parameters, which are linear functions of the fitted voxel-level residue, ultimately scale with the magnitude of the time-course data. Mean transit time and extraction fractions deviate somewhat from this pattern. This is likely to be related to the fact that both the mean transit time and extraction fraction are defined in terms of ratios of the *V*_d_, *K*_d_, and *K_i_* variables and, as a result, do not necessarily scale with the scale of the voxel time course. The large blood vessels are seen to impact the structure of the MIP uncertainty for several parameters. The algorithms developed allow kinetic mapping, including the bootstrapping process, to be achieved in a timely fashion. On a single 3.2-GHz processor, the compute time for the NPRM kinetic analysis including the definition of the DGP is 140 min; each bootstrap replicate took 80 min.

### Statistical Analysis

#### Representation of VOI Time-Course Data

**FIGURE 3.**
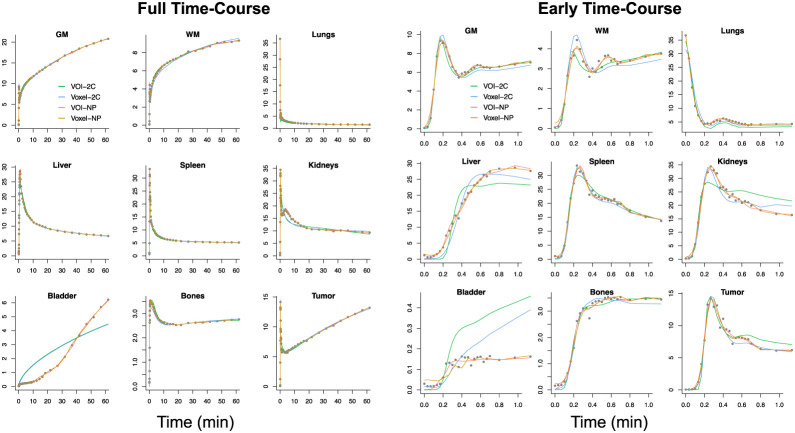
Results of alternative fitting of VOI data used in Figure 2. Data are points, and line colors correspond to methods used. Full time course is on left; first minute is on right. GM = gray matter; NP = nonparametric; WM = white matter.

The full time course as well as the time course over the first minute of data acquisition are shown in Figure 3. Average VOI time-course data are fit directly using the nonparametric and 2C models; averages of voxel-level fits are also provided. This gives a reference to the results reported previously (2). Although the 2C fitting of some VOIs is reasonable, for example, gray and white matter, there are clearly some VOIs where 2C modeling is substantially inferior (e.g., kidney, liver, bone, and bladder). The data fit achieved by the VOI averaging of the voxel-level nonparametric fit is quite good overall and especially over the first 1 min of acquisition. However, it is important to appreciate that almost half of the total number of frames occur in the first 80 s. For this example, over the first minute, differences between the VOI average of the voxelwise 2C fits and the fit of the 2C model to the mean of the VOI time-course data are quite pronounced. In contrast, differences between the corresponding nonparametric fits are much smaller.

Quantitative summaries of the nonparametric fitting of VOI time-course data and comparisons with direct analysis of the mean VOI time-course data using nonparametric and 2C analysis are presented in Table 1. Although values from the weighted-residual-sums-of-squares fit for VOIs are similar based on the VOI average of voxel-level nonparametric fits or by direct fitting of the VOI time-course data, there is a marked increase in weighted-residual-sums-of-squares fits when the VOI time course is approximated using the best-fitting 2C model. VOI time-course fitting by the nonparametric model is consistently improved by averaging voxel-level nonparametric fits; the percent improvement is a modest 50%. VOI time-course fitting by the 2C model is substantially worse than the nonparametric fitting. The mean percent improvement here is almost 390%.

**TABLE 1.** VOI Time-Course Fitting Across 24 Studies

Region	Size (×10^3^ voxels)*	Voxel-NP	VOI-NP	VOI-2C	Voxel-NP vs. VOI-NP	Voxel-NP vs. VOI-2C
GM	122.5 ± 23.3	0.02 ± 0.06	0.04 ± 0.14	0.09 ± 0.26	35 ± 50	161 ± 216
WM	18.8 ± 7.0	0.02 ± 0.04	0.02 ± 0.02	0.06 ± 0.20	25 ± 32	141 ± 117
Lung	434.5 ± 124.2	0.06 ± 0.27	0.08 ± 0.69	0.10 ± 0.29	70 ± 76	448 ± 690
Liver	233.0 ± 66.1	0.08 ± 0.08	0.07 ± 0.08	0.55 ± 0.30	46 ± 39	865 ± 453
Spleen	42.8 ± 48.5	0.10 ± 0.11	0.11 ± 0.16	0.13 ± 0.09	69 ± 40	64 ± 87
Kidney	40.5 ± 10.7	0.27 ± 0.26	0.35 ± 0.56	1.83 ± 1.29	83 ± 45	880 ± 576
Bladder	234.9 ± 81.6	0.04 ± 0.10	0.07 ± 0.77	3.25 ± 3.04	887 ± 783	18,266 ± 14,809
Bones	306.2 ± 82.7	0.002 ± 0.003	0.002 ± 0.003	0.007 ± 0.01	35 ± 23	327 ± 206
Tumor	2.5 ± 7.4	0.21 ± 0.25	0.23 ± 0.51	0.67 ± 3.77	28 ± 31	231 ± 250

* 1 voxel = 1.65 × 1.65 × 1.65 mm^3^.

Mean *±* SD of weighted residual sums of squares (WRSS) deviations between VOI time course and VOI average of voxelwise (voxel-NP), direct NP (VOI-NP), and 2C (VOI-2C) fits are shown. Last 2 columns show mean *±* SD of percent deviations between WRSS of voxel-NP and VOI-NP fits and also between voxel-NP and VOI-2C values.

GM = gray matter; WM = white matter.

#### VOI Kinetics

VOI kinetics are reported in Table 2. Statistically significant deviations between the kinetics recovered by alternative methods are largely linked to early time-course parameters (Fig. 1), particularly for *V*_b_. Deviations between voxel-averaged parameters and values recovered from nonparametric and 2C analysis of the VOI time course are much smaller for nonparametric analysis than for 2C analysis. However, it is noteworthy that, for most VOIs, *K_i_* is quite similar in magnitude across all 3 analyses. This might be because flux is a late-time-course parameter (Fig. 1), and alternative methods fit the late time course quite similarly (Fig. 3).

**TABLE 2.** VOI Kinetics Recovered Using Different Methodologies

Method	Region	*V*_b_ (mL/g)	*V*_d_ (mL/g)	*K*_d _(mL/min/g)	*K_i_* (mL/min/100 g)	MTT (min)	Ext (%)
Voxel-NP	GM	0.05 ± 0.01	0.88 ± 0.26	0.16 ± 0.03	3.01 ± 0.81	5.80 ± 1.64	18.38 ± 5.18
	WM	0.03 ± 0.01	0.64 ± 0.27	0.10 ± 0.03	1.09 ± 0.33	6.61 ± 2.04	12.30 ± 4.12
	Lung	0.18 ± 0.04	0.09 ± 0.03	0.03 ± 0.01	0.07 ± 0.04	3.17 ± 0.77	3.17 ± 1.82
	Liver	0.09 ± 0.04	0.86 ± 0.08	0.54 ± 0.11	0.23 ± 0.08	1.77 ± 0.41	0.57 ± 0.38
	Spleen	0.21 ± 0.09	0.42 ± 0.09	0.41 ± 0.14	0.23 ± 0.16	1.39 ± 0.54	1.14 ± 2.09
	Kidney	0.25 ± 0.07	1.20 ± 0.34	0.49 ± 0.11	0.42 ± 0.25	2.62 ± 0.60	1.22 ± 0.97
	Bladder	0.00 ± 0.01	0.54 ± 0.32	0.04 ± 0.03	1.46 ± 1.13	6.73 ± 2.85	19.11 ± 11.36
	Bones	0.03 ± 0.02	0.22 ± 0.07	0.08 ± 0.02	0.27 ± 0.08	3.54 ± 0.79	4.66 ± 1.32
	Tumor	0.08 ± 0.05	0.65 ± 0.38	0.19 ± 0.08	2.33 ± 1.59	3.62 ± 1.57	12.93 ± 6.44
VOI-NP	GM	0.04 ± 0.01*	0.58 ± 0.16*	0.13 ± 0.03*	3.25 ± 0.89*	4.53 ± 1.19*	20.13 ± 5.42
	WM	0.02 ± 0.01*	0.58 ± 0.31^†^	0.09 ± 0.03	1.10 ± 0.38	5.99 ± 2.25^†^	10.96 ± 4.37
	Lung	0.17 ± 0.04^†^	0.08 ± 0.03^‡^	0.03 ± 0.02	0.08 ± 0.04^†^	2.43 ± 0.65*	2.66 ± 1.48
	Liver	0.05 ± 0.04*	0.83 ± 0.07^‡^	0.53 ± 0.13	0.28 ± 0.08*	1.64 ± 0.39*	0.57 ± 0.24
	Spleen	0.18 ± 0.09^†^	0.39 ± 0.11^‡^	0.46 ± 0.17^‡^	0.29 ± 0.19*	0.91 ± 0.22*	0.86 ± 1.38^‡^
	Kidney	0.23 ± 0.08	1.19 ± 0.37	0.51 ± 0.15	0.30 ± 0.26^‡^	2.46 ± 0.82	0.62 ± 0.51*
	Bladder	0.00 ± 0.01*	0.44 ± 0.29^†^	0.03 ± 0.03*	1.61 ± 1.21^†^	13.85 ± 11.15*	33.87 ± 26.48*
	Bones	0.01 ± 0.01*	0.17 ± 0.08*	0.08 ± 0.03	0.29 ± 0.07*	2.24 ± 0.50*	3.80 ± 0.90^†^
	Tumor	0.07 ± 0.04*	0.45 ± 0.34*	0.18 ± 0.09^‡^	2.48 ± 1.62*	2.62 ± 1.59*	13.27 ± 7.68
VOI-2C	GM	0.03 ± 0.01*	0.56 ± 0.23	0.09 ± 0.02*	3.12 ± 0.87^†^	5.91 ± 1.71^‡^	26.41 ± 6.41*
	WM	0.02 ± 0.01*	0.55 ± 0.16	0.05 ± 0.01*	1.13 ± 0.32	11.00 ± 3.23*	18.65 ± 4.48*
	Lung	0.15 ± 0.04*	0.09 ± 0.03^‡^	0.04 ± 0.01^†^	0.07 ± 0.04^‡^	1.18 ± 1.44*	1.70 ± 0.83*
	Liver	0.02 ± 0.01*	0.88 ± 0.09*	0.53 ± 0.13	0.23 ± 0.08*	1.68 ± 0.33	0.45 ± 0.21*
	Spleen	0.11 ± 0.05*	0.49 ± 0.12*	0.61 ± 0.18*	0.21 ± 0.21*	0.73 ± 0.32*	0.69 ± 1.97*
	Kidney	0.15 ± 0.05*	1.25 ± 0.36^‡^	0.42 ± 0.11^†^	0.21 ± 0.22^‡^	2.81 ± 1.18^‡^	0.52 ± 0.54
	Bladder	0.00 ± 0.00	0.05 ± 0.10*	0.01 ± 0.02*	1.63 ± 0.95	1.66 ± 5.36*	86.89 ± 29.33*
	Bones	0.00 ± 0.00*	0.20 ± 0.06^†^	0.08 ± 0.03	0.26 ± 0.07*	2.65 ± 0.65*	3.53 ± 0.94^‡^
	Tumor	0.05 ± 0.05*	0.34 ± 0.19	0.17 ± 0.10	2.38 ± 1.50^‡^	1.80 ± 1.26^†^	16.83 ± 18.38

* *P* < 0.001.

† *P* < 0.01.

‡ *P* < 0.05.

MTT = mean transit time; Ext = extraction fraction; voxel-NP = VOI-averaged voxel kinetics; GM = gray matter; WM = white matter; VOI-NP = VOI time course kinetics obtained by NP; VOI-2C = VOI time-course kinetics obtained by 2C.

Values are mean *±* SD.

#### DGP Model

**FIGURE 4.**
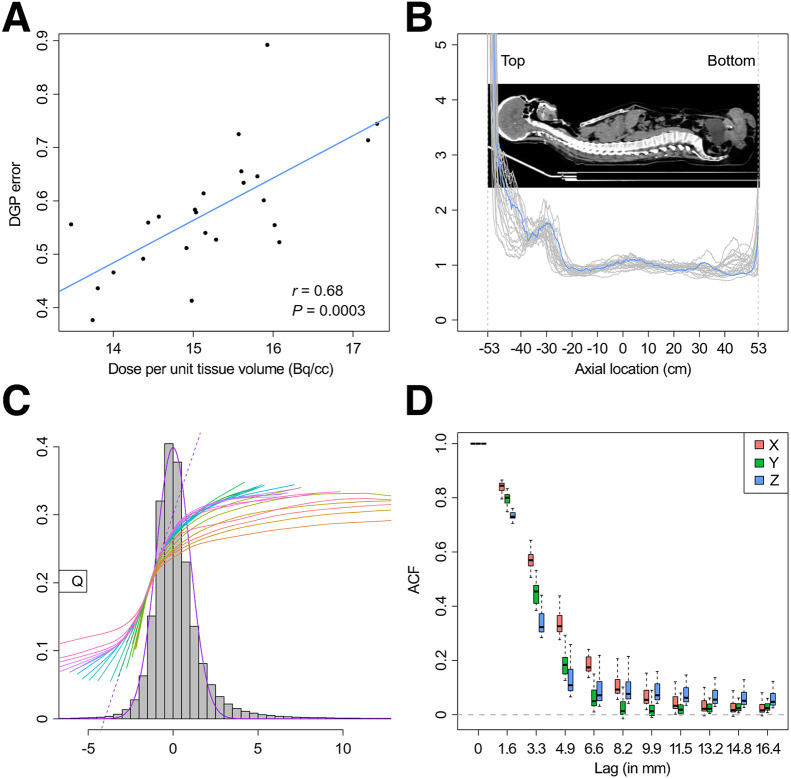
Bootstrap DGP from Equation 2: DGP scale (${\hat{\sigma }}_{e}$) vs. injected dose per unit tissue voxel (A), axially averaged scale (B), error distribution (histogram) with comparison to standard gaussian (purple line) (C), and box plot of directional (*x*, perpendicular to scan table; *z*, axial) autocorrelation function (ACF) across all studies (D).

Figure 4 and Supplemental Figure 2 show an expected linear relation between the scale of the DGP and study dose; the linear correlation of 0.68 is highly significant. The axially averaged spatial scale of the DGP increases toward the top and bottom of the patient in the FOV. As expected, the increased scale is not just a function of the nominal sensitivity but is clearly impacted by patient-specific factors including the varying uptake, attenuation, and perhaps any impacts of small patient movements. The skewed nature of random fluctuations in the DGP model, which vary on the basis of the data coefficient of variation, are fully consistent with patterns for iteratively reconstructed PET data (10,21). The full width at half maximum of the autocorrelation functions in each direction is on the order of 2–3 mm. The coordinatewise autocorrelation functions show greater spatial persistence in the *x* (perpendicular to scanning bed) and *z* (axially) directions (Supplemental Fig. 3). This could align with involuntary patient movements during scanning.

#### SEs of VOI Kinetics

**FIGURE 5.**
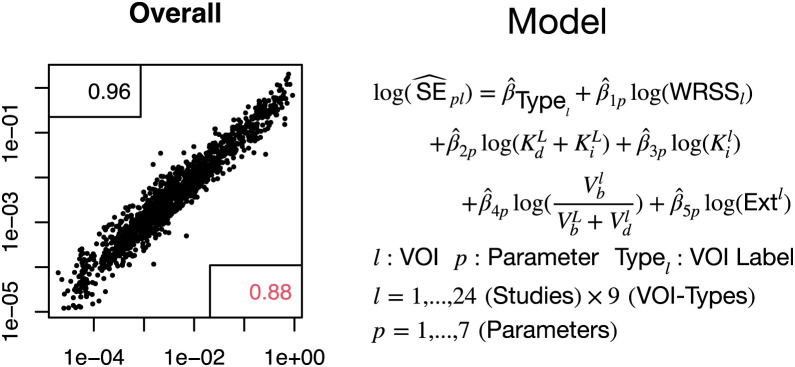
Prediction of VOI kinetic SEs (vertical axis) via log-linear model prediction ($\hat{\text{SE}}$, horizontal axis) formula indicated. Correlation for logarithmic SE value is 0.96 and for raw scale is 0.88. WRSS = weighted residual sums of squares; Ext = extraction fraction.

SEs of VOI kinetics (voxel-level nonparametric) are well approximated using a log-linear model that accounts for the VOI type, the VOI mean kinetics, and the residual weighted root mean square error of the voxel-level nonparametric fit of the VOI time course (Fig. 5; Supplemental Fig. 4). The overall correlation between the bootstrap-measured SE and the SE values predicted by log-linear modeling is 0.96, which is also quite high for individual kinetic parameters.

## DISCUSSION

This work demonstrates the practicality of using image-domain bootstrapping for the construction of patient-specific uncertainty assessment in kinetics variables for voxel, VOI, and more complex derived quantities such as MIPs from a whole-body dynamic ^18^F-FDG PET study. This development creates an opportunity to incorporate uncertainty about a PET-derived kinetic biomarker that might be used to guide a clinical decision for a patient. This could be particularly helpful in cases where the biomarker value is close to a boundary between alternative treatment options.

**FIGURE 6.**
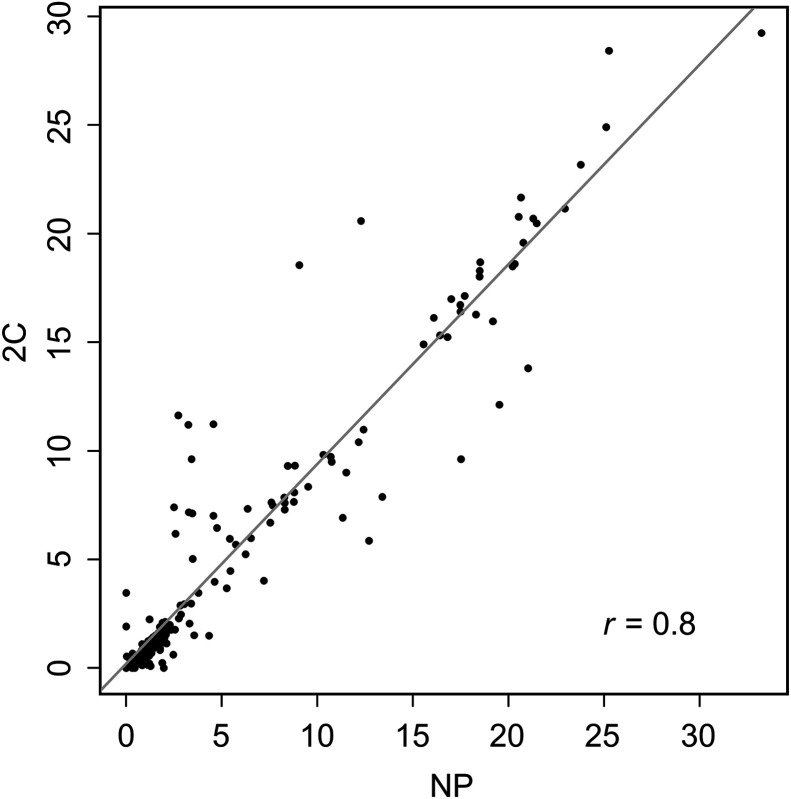
Overall relation between ^18^F-FDG MR *K_i_* computed using nonparametric (NP) voxel (horizontal axis) and 2C (vertical axis) analysis. Best-fit linear regression is shown as solid line.

Bootstrap reliability depends both on the number of bootstrap simulations (*N*_B_) used and on the accuracy of the representation of the data used in the DGP (20). Computational resources dictate the choice of *N*_B_. The results here are based on just an *N*_B_ of 25, but for the data in Figure 2, a 4-fold increase in *N*_B_ leads to little qualitative change in derived voxel-level SE (Supplemental Fig. 5). Table 1 clearly demonstrates the benefit of using a nonparametric methodology in the DGP. Relative to the well-established 2C ^18^F-FDG model, substantial and highly significant improvements in data representation are achieved using the nonparametric approach. These benefits are mostly associated with the ability of the nonparametric technique to capture the highly resolved early time-course pattern of data from the current generation of PET scanners. The generally more modest deviations between nonparametric and 2C fits beyond the early time period, say after 1 min, suggest that the deficiencies in the 2C model may primarily relate to the lack of sophistication in the representation of the vascular components of blood–tissue exchange (22). The high temporal resolution of the scans here, as well as the use of a bolus injection, contributes to the ability to scrutinize the 2C model in ways that have likely not been possible in the past. The VOIs here are large and heterogeneous—far from the assumption of homogeneous well-mixed compartments that underly the 2C model. However, it is notable that our previous work (23) reported significant discrepancies between 2C and nonparametric representation of dynamic ^18^F-FDG brain data in healthy subjects using much smaller and highly homogeneous VOIs. Similar to what is reported in Table 2 for gray and white matter, the discrepancies primarily impact the accuracy of the initial phase of the ^18^F-FDG tissue residue—*V*_b_ especially—but have much less impact on several other variables including flux and *V*_d_. However, statistically significant differences between voxel nonparametric and VOI 2C parameters do not imply that parameters are unrelated. For example, Figure 6 and Supplemental Figure 6 show pairwise plots and summary correlations for the ^18^F-FDG metabolic rate (MR) flux scaled by the plasma glucose in Equation 4. The strong linear dependence in Figure 6 emphasizes the importance of differentiating statistical and practical significance. Calculated *K_i_* based on nonparametric or 2C analysis would likely yield similarly effective diagnostic values. Indeed, it is well appreciated that even simpler assessments of ^18^F-FDG flux by Patlak analysis and SUV are also highly effective.

The nonparametric technique here uses a linear basis, but the structure and number of elements involved are adapted to the full 4-dimensional dynamic data and guided by cross validation to prevent overfitting (10). The accuracy and stability of a kinetic mapping procedure are best evaluated numerically, which was reported previously (24)—studies based on a 2-min constant infusion injection of ^18^F-FDG and a temporal sampling protocol in which the shortest time frames were 20 s in duration, providing mean-square-error performance characteristics of NPRM and 2C kinetic mapping of ^18^F-FDG PET data as a function of the study dose and as a function of whether the underlying ground truth is governed by a compartmental model or not. In this study, the accuracy of the flux is largely unaffected by whether a 2C or an NPRM mapping technique is used. Across other kinetic variables, when the ground truth is noncompartmental, the NPRM approach is much better. Remarkably, when the ground truth is a 2C model, the NPRM continues to outperform the 2C approach, especially for variables such as *V*_b_ and *V*_d_. Further study of the mean-square-error performance would clearly be useful, particularly in settings where the ground truth, study protocol, and scanning methods are similar to those encountered with the current generation of whole-body ^18^F-FDG PET studies.

VOI values of 3 variables, FDG metabolic rate (MR_FDG_), distribution volume (DV), and vascular blood flow (BF), are compared with literature reports. Each variable is directly obtained by simple scaling of our summary kinetic values: *K_i_*, *V*_d_, and *V*_b_.

$${\text{MR}}_{\text{FDG}}={\text{μ}}_{\text{glc}}K_{i};\text{ DV}=V_{\text{d}}\text{; BF}=\frac{V_{\text{b}}}{t^{*}/2}.$$ Eq. 4

Here, ${\text{μ}}_{\text{glc}}$ is the plasma glucose concentration and t* is the value used to define the vascular component in the decomposition of the Meier–Zierler residue in Figure 1. In a cancer setting, ^18^F-FDG MR is by far the most clinically important of these variables. Note that we do not try to use ^18^F-FDG as a means to evaluate the glucose MR, as described in Phelps et al. (17). Barrio et al. (6) expressed considerable doubt on the ability to do this in the context of cancer applications. Consideration of the *V*_b_ variable is motivated by interest in deriving potentially useful additional diagnostic information related to tissue vascularity from ^18^F-FDG (1,25,26). There is no intention of questioning PET ^15^O-H_2_O as the gold standard for *V*_b_ determination. Our *V*_b_ formula is an application of the central volume theorem (14) based on an assumed mean transit time in the vasculature of t*/2 (here, 7.5 s) for the collection of tracer atoms whose tissue transit time in the local voxel is less than 15 s.

Table 3 compares the VOI averages of 3 variables to those in the literature. For ^18^F-FDG MR and *V*_d_, the values are seen to be in the range reported using 2C and Patlak analyses (27). *V*_b_ values are compared with those in reports based on PET ^15^O-H_2_O and dynamic susceptibility contrast MR techniques. The results for the NPRM approach are remarkably similar to those in the literature, particularly given that the study group here is older and unhealthy (28). Further examination of the *V*_b_ variable could be merited. Viability of conducting PET ^15^O-H_2_O on this scanner was previously demonstrated (29). Note that some of the deviation in Table 3 may be related to scaling differences between the use of whole-blood activity as an AIF (like ours) and other analyses that used the arterial plasma activity time course as an AIF.

**TABLE 3.** Comparison with Literature Values for ^18^F-FDG MR, *V*_d_, and *V*_b_ in Different Tissues from Equation 4

Parameter	Method	GM	WM	Lung	Liver	Spleen	Kidney	Bones
^18^F-FDG MR (μmol/100 g/min)	Voxel-NP	18.54 ± 6.81	6.66 ± 2.44	0.42 ± 0.31	1.40 ± 0.53	1.40 ± 0.94	2.52 ± 1.51	1.63 ± 0.63
	VOI-2C*	18.98 ± 7.18	6.83 ± 2.26	0.43 ± 0.32	1.38 ± 0.47	1.08 ± 1.24	1.19 ± 1.43	1.58 ± 0.55
		20.26 ± 6.14	7.17 ± 2.09	0.47 ± 0.35	1.39 ± 0.89	1.39 ± 0.83	1.98 ± 2.20	1.72 ± 0.63
		22.22 ± 2.71	7.60 ± 1.58	0.60 ± 0.42	5.22 ± 2.67	9.40 ± 4.57	9.15 ± 6.44	1.34 ± 0.46
		17.46 [11.68–27.61]	6.03 [4.02–9.53]	0.35 [0.03–1.74]	2.02 [0.74–4.35]	2.45 [1.18–15.30]	3.81 [0.08–7.95]	3.69 [1.08–9.09]
*V*_d_ (mL/g)	Voxel-NP	0.88 ± 0.26	0.64 ± 0.27	0.09 ± 0.03	0.86 ± 0.08	0.42 ± 0.09	1.20 ± 0.34	0.22 ± 0.07
	VOI-2C^†^	0.56 ± 0.23	0.55 ± 0.16	0.09 ± 0.03	0.88 ± 0.09	0.49 ± 0.12	1.25 ± 0.36	0.20 ± 0.06
		0.62 ± 0.56	0.67 ± 0.22	0.26 ± 0.06	0.98 ± 0.15	0.63 ± 0.14	1.44 ± 0.55	0.23 ± 0.10
		0.81 [0.14–1.41]	0.46 [0.15–0.87]	0.15 [0.02–0.28]	0.84 [0.44–1.26]	0.58 [0.10–1.12]	0.96 [0.33–1.41]	0.31 [0.04–0.51]
*V*_b_ (mL/min/g)	Voxel-NP	0.43 ± 0.11	0.24 ± 0.08	1.41 ± 0.32	0.73 ± 0.34	1.70 ± 0.74	1.99 ± 0.58	0.22 ± 0.12
	VOI-2C	0.26 ± 0.04	0.13 ± 0.04	1.16 ± 0.34	0.14 ± 0.07	0.85 ± 0.42	1.22 ± 0.38	0.03 ± 0.02
		0.59 ± 0.11 (32)	0.20 ± 0.04 (32)	1.40 ± 0.30 (33)	1.11 ± 0.34 (34)	1.92 ± 0.76 (35)	1.74 ± 0.44 (35)	0.18 ± 0.03 (36)
		0.41 ± 0.11 (37)	0.22 ± 0.04 (37)	1.21 ± 0.32 (38)	1.78 ± 0.56 (39)	1.68 ± 0.12 (40)	1.57 ± 0.60 (41)	0.18 ± 0.05 (42)

* References 2,4,27,30,31.

† References 2,27.

GM = gray matter; WM = white matter; Voxel-NP = VOI-averaged voxel kinetics; VOI-2C = VOI time course kinetics obtained by 2C analysis.

Data are mean *±* SD, or median followed by range in brackets.

**FIGURE 7.**
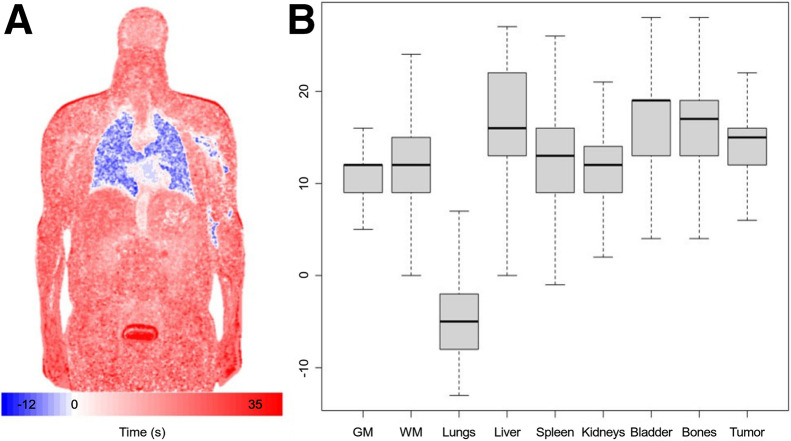
(A) Delay image corresponding to coronal CT slice in Figure 2; values are amplitude-weighted delay values, $\left\{\text{δ}+\Delta_{k},k=1,2,\ldots ,K\right\}$, in Equation 1. Data are centered so that mean delay in descending aorta is 0. (B) Box plots of distribution of mapped delay values in seconds by VOI. GM = gray matter; WM = white matter.

Although our focus has been on parameters that have traditionally been used to quantify ^18^F-FDG PET dynamics, the nonparametric technique provides a possibility to also evaluate a summary of the arrival pattern of ^18^F-FDG at the voxel level. A sample amplitude-weighted average of voxel-level basis element delay as shown in Equation 1 is shown in Figure 7. There is early arrival of the signal to the lung and much more delayed arrival to the bladder and more peripheral regions (1). More detailed consideration of the ^18^F-FDG arrival pattern may be worthwhile.

## CONCLUSION

NPRM kinetic analysis together with bootstrap assessment of uncertainty is practically feasible in the context of large-scale long-axial-FOV ^18^F-FDG PET data. This provides an ability to incorporate patient-specific uncertainty measures of kinetic biomarkers recovered from dynamic PET to support clinical decisions.

## DISCLOSURE

This research is supported by Science Foundation Ireland grant PI-11/1027 and by the National Cancer Institute USA grant R33-CA225310. Kuangyu Shi and Axel Rominger are funded by Siemens Healthineers and Novartis. Hasan Sari is an employee of Siemens Healthineers. No other potential conflict of interest relevant to this article was reported.

## Notes

KEY POINTS **QUESTION:** Is it feasible to map kinetics together with uncertainty in long-axial-FOV dynamic ^18^F-FDG PET studies? **PERTINENT FINDINGS:** NRPM analysis together with image-domain bootstrapping is a suitable methodology for mapping kinetics. **IMPLICATIONS FOR PATIENT CARE:** The ability to derive uncertainties in complex kinetic biomarkers could enhance patient-specific decision-making for guiding treatment of cancer patients.

## References

[1] Feng T Zhao Y Shi H. Total-body quantitative parametric imaging of early kinetics of ^18^F-FDG. J Nucl Med. 2021;62:738–744. 32948679 10.2967/jnumed.119.238113 PMC8844261

[2] Sari H Mingels C Alberts I. First results on kinetic modelling and parametric imaging of dynamic ^18^F-FDG datasets from a long axial FOV PET scanner in oncological patients. Eur J Nucl Med Mol Imaging. 2022;49:1997–2009. 34981164 10.1007/s00259-021-05623-6

[3] Wang G Nardo L Parikh M. Total-body PET multiparametric imaging of cancer using a voxelwise strategy of compartmental modeling. J Nucl Med. 2022;63:1274–1281. 34795014 10.2967/jnumed.121.262668 PMC9364337

[4] Spence AM Muzi M Graham MM. Glucose metabolism in human malignant gliomas measured quantitatively with PET, 1-[C-11] glucose and FDG: analysis of the FDG lumped constant. J Nucl Med. 1998;39:440–448. 9529289

[5] Muzi M Spence AM O’Sullivan F. Kinetic analysis of 3′-deoxy-3′-^18^F-fluorothymidine in patients with gliomas. J Nucl Med. 2006;47:1612–1621. 17015896

[6] Barrio JR Huang SC Satyamurthy N. Does 2-FDG PET accurately reflect quantitative in vivo glucose utilization? J Nucl Med. 2020;61:931–937. 31676728 10.2967/jnumed.119.237446 PMC7818046

[7] Gu F Wu Q. Quantitation of dynamic total-body PET imaging: recent developments and future perspectives. Eur J Nucl Med Mol Imaging. 2023;50:3538–3557. 37460750 10.1007/s00259-023-06299-w PMC10547641

[8] Cunningham VJ Jones T. Spectral analysis of dynamic PET studies. J Cereb Blood Flow Metab. 1993;13:15–23. 8417003 10.1038/jcbfm.1993.5

[9] O’Sullivan F. Imaging radiotracer model parameters in PET: a mixture analysis approach. IEEE Trans Med Imaging. 1993;12:399–412. 18218432 10.1109/42.241867

[10] O’Sullivan F Gu F Wu Q O’Suilleabhain LD. A generalized linear modeling approach to bootstrapping multi-frame PET image data. Med Image Anal. 2021;72:102132. 34186431 10.1016/j.media.2021.102132 PMC8717713

[11] Gu F Wu Q O’Sullivan F. Image-domain bootstrapping of PET time-course data for assessment of uncertainty in complex regional summaries of mapped kinetics. IEEE Xplore website. https://ieeexplore.ieee.org/document/9875531. Published September 9, 2022. Accessed March 22, 2024.

[12] Naganawa M Gallezot JD Shah V. Assessment of population-based input functions for Patlak imaging of whole body dynamic ^18^F-FDG PET. EJNMMI Phys. 2020;7:67. 33226522 10.1186/s40658-020-00330-x PMC7683759

[13] Prenosil GA Sari H Fürstner M. Performance characteristics of the Biograph Vision Quadra PET/CT system with a long axial field of view using the NEMA NU 2-2018 standard. J Nucl Med. 2022;63:476–484. 34301780 10.2967/jnumed.121.261972

[14] Meier P Zierler KL. On the theory of the indicator-dilution method for measurement of blood flow and volume. J Appl Physiol. 1954;6:731–744. 13174454 10.1152/jappl.1954.6.12.731

[15] Patlak CS Blasberg RG Fenstermacher JD. Graphical evaluation of blood-to-brain transfer constants from multiple-time uptake data. J Cereb Blood Flow Metab. 1983;3:1–7. 6822610 10.1038/jcbfm.1983.1

[16] Kety SS Schmidt CF. The determination of cerebral blood flow in humans by the use of nitrous oxide in low concentrations. Am J Physiol. 1945;143:53–66.

[17] Phelps ME Huang SC Hoffman EJ Selin C Sokoloff L Kuhl DE. Tomographic measurement of local cerebral glucose metabolic rate in humans with (F-18)2-fluoro-2-deoxy-D-glucose: validation of method. Ann Neurol. 1979;6:371–388. 117743 10.1002/ana.410060502

[18] O’Sullivan F Muzi M Mankoff DA Eary JF Spence AM Krohn KA. Voxel-level mapping of tracer kinetics in PET studies: a statistical approach emphasizing tissue life tables. Ann Appl Stat. 2014;8:1065–1094. 25392718 10.1214/14-aoas732 PMC4225726

[19] Haynor DR Woods SD. Resampling estimates of precision in emission tomography. IEEE Trans Med Imaging. 1989;8:337–343. 18230533 10.1109/42.41486

[20] Bradley E Tibshirani RJ. An Introduction to the Bootstrap. CRC Press, 1994.

[21] Mou T Huang J O’Sullivan F. The gamma characteristic of reconstructed PET images: implications for ROI analysis. IEEE Trans Med Imaging. 2018;37:1092–1102. 29727273 10.1109/TMI.2017.2770147

[22] Li Z Yipintsoi T Bassingthwaighte JB. Nonlinear model for capillary-tissue oxygen transport and metabolism. Ann Biomed Eng. 1997;25:604–619. 9236974 10.1007/bf02684839 PMC3589573

[23] O’Sullivan F Mark M Spence AM. Nonparametric residue analysis of dynamic PET data with application to cerebral FDG studies in normals. J Am Stat Assoc. 2009;104:556–571. 19830267 10.1198/jasa.2009.0021 PMC2760850

[24] Gu F O’Sullivan F Muzi M Mankoff DA. Quantitation of multiple injection dynamic PET scans: an investigation of the benefits of pooling data from separate scans when mapping kinetics. Phys Med Biol. 2021.66:135010. 10.1088/1361-6560/ac0683 PMC8284854 34049293

[25] Pouzot C Richard JC Gros A. Noninvasive quantitative assessment of pulmonary blood flow with ^18^F-FDG PET. J Nucl Med. 2013;54:1653–1660. 23907755 10.2967/jnumed.112.116699

[26] Mullani NA Herbst RS O’Neil RG Gould KL Barron BJ Abbruzzese JL. Tumor blood flow measured by PET dynamic imaging of first-pass ^18^F-FDG uptake: a comparison with ^15^O-labeled water-measured blood flow. J Nucl Med. 2008;49:517–523. 18344436 10.2967/jnumed.107.048504

[27] Dias AH Hansen AK Munk OL Gormsen LC. Normal values for ^18^F-FDG uptake in organs and tissues measured by dynamic whole body multiparametric FDG PET in 126 patients. EJNMMI Res. 2022;12:15. 35254514 10.1186/s13550-022-00884-0 PMC8901901

[28] Wu C Honarmand AR Schnell S. Age-related changes of normal cerebral and cardiac blood flow in children and adults aged 7 months to 61 years. J Am Heart Assoc. 2016;5:e002657. 26727967 10.1161/JAHA.115.002657 PMC4859381

[29] Knuuti J Tuisku J Kärpijoki H. Quantitative perfusion imaging with total-body PET. J Nucl Med. 2023;64(suppl 2):11S–19S. 37918848 10.2967/jnumed.122.264870

[30] Liu G Xu H Hu P. Kinetic metrics of ^18^F-FDG in normal human organs identified by systematic dynamic total-body positron emission tomography. Eur J Nucl Med Mol Imaging. 2021;48:2363–2372. 33416959 10.1007/s00259-020-05124-y

[31] Graham MM Mark M Spence AM. The FDG lumped constant in normal human brain. J Nucl Med. 2002;43:1157–1166. 12215553

[32] Huang SC Carson RE Hoffman EJ. Quantitative measurement of local cerebral blood flow in humans by positron computed tomography and ^15^O-water. J Cereb Blood Flow Metab. 1983;3:141–153. 6601663 10.1038/jcbfm.1983.21

[33] Matsunaga K Yanagawa M Otsuka T. Quantitative pulmonary blood flow measurement using ^15^O-H_2_O PET with and without tissue fraction correction: a comparison study. EJNMMI Res. 2017;7:102. 29274016 10.1186/s13550-017-0350-8 PMC5741573

[34] Slimani L Kudomi N Oikonen V. Quantification of liver perfusion with [^15^O]H_2_O-PET and its relationship with glucose metabolism and substrate levels. J Hepatol. 2008;48:974–982. 18384905 10.1016/j.jhep.2008.01.029

[35] Lauritsen KM Søndergaard E Luong TV Møller N Gormsen LC. Acute hyperketonemia does not affect glucose or palmitate uptake in abdominal organs or skeletal muscle. J Clin Endocrinol Metab. 2020;105:1785–1790. 10.1210/clinem/dgaa122 32161953

[36] Kahn D Weiner GJ Ben-Haim S. Positron emission tomographic measurement of bone marrow blood flow to the pelvis and lumbar vertebrae in young normal adults. Blood. 1994;83:958–963. 8111065

[37] Markus HS Lythgoe DJ Ostegaard L O’Sullivan M Williams SC. Reduced cerebral blood flow in white matter in ischaemic leukoaraiosis demonstrated using quantitative exogenous contrast based perfusion MRI. J Neurol Neurosurg Psychiatry. 2000;69:48–53. 10864603 10.1136/jnnp.69.1.48 PMC1737001

[38] Schuster DP Kaplan JD Gauvain K Welch MJ Markham J. Measurement of regional pulmonary blood flow with PET. J Nucl Med. 1995;36:371–377. 7884497

[39] Materne R Van Beers E. Non-invasive quantification of liver perfusion with dynamic computed tomography and a dual-input one-compartmental model. Clin Sci (Lond). 2000;99:517–525. 11099395

[40] Oguro A Taniguchi H Koyama H. Quantification of human splenic blood flow: quantitative measurement of splenic blood flow with H_2_ ^15^O and a dynamic state method. Ann Nucl Med. 1993;7:245–250. 8292450 10.1007/BF03164705

[41] Kudomi N Koivuviita N Liukko KE. Parametric renal blood flow imaging using [^15^O]H_2_O and PET. Eur J Nucl Med Mol Imaging. 2009;36:683–691. 19050876 10.1007/s00259-008-0994-8

[42] Piert M Machulla H-J Jahn M Stahlschmidt A Becker GA Zittel TT. Coupling of porcine bone blood flow and metabolism in high-turnover bone disease measured by [^15^O]H_2_O and [^18^F]fluoride ion positron emission tomography. Eur J Nucl Med Mol Imaging. 2002;29:907–914. 12111131 10.1007/s00259-002-0797-2

